# Purification Process of a Recombinant Human Follicle Stimulating Hormone Biosimilar (Primapur^®^) to Yield a Pharmaceutical Product with High Batch-to-Batch Consistency

**DOI:** 10.3390/pharmaceutics14010096

**Published:** 2022-01-01

**Authors:** Maria Sinegubova, Ivan Vorobiev, Anatoly Klishin, Dmitry Eremin, Nadezhda Orlova, Natalya Orlova, Mikhail Polzikov

**Affiliations:** 1 Laboratory of Mammalian Cell Bioengineering, Institute of Bioengineering, Federal State Institution «Federal Research Centre «Fundamentals of Biotechnology» of the Russian Academy of Sciences», Leninsky Prospect, 33, Build. 2, 119071 Moscow, Russia; ptichman@gmail.com (I.V.); nobiol@gmail.com (N.O.); 2 IVFarma LLC, Nauchnyi Proezd, 20, Build. 2, 117246 Moscow, Russia; sciencetank@gmail.com; 3 State Research Institute of Genetics and Selection of Industrial Microorganisms of National Research Center «Kurchatov Institute», Dorozhniy Proezd, 1, 117545 Moscow, Russia; anatolii-klishin@bk.ru (A.K.); orlova.chemist@gmail.com (N.O.)

**Keywords:** biopharmaceuticals, biosimilars, recombinant human follicle stimulating hormone (r-hFSH), follitropin alpha, downstream, immunoaffinity chromatography, dynamic binding capacity (DBC), batch-to-batch consistency

## Abstract

Recombinant human follicle stimulating hormone (r-hFSH) is widely used for infertility treatment and is subject to the development of biosimilars. There are different purification strategies that can yield r-hFSH of pharmaceutical quality from Chinese hamster ovary cell culture broth. We developed a purification process for r-hFSH centered on immunoaffinity chromatography with single-domain recombinant camelid antibodies. The resulting downstream process is simple and devoid of ultrafiltration operations. Studies on chromatography resin resource and ligand leakage showed that the immunoaffinity matrix employed was suitable for industrial use and stable for at least 40 full chromatography cycles, and the leaked single-domain antibody ligand was completely removed by subsequent purification steps. All chromatography resins employed withstood the same 40 cycles of use without significant changes in separation efficiency and product binding capacity. The resulting industrial purification process yielded batches of r-hFSH with consistent levels of purity and bioactivity.

## 1. Introduction

Follicle-stimulating hormone (FSH) is a heterodimeric glycoprotein of the gonadotropin family, consisting of two non-covalently associated subunits (chains). The alpha-subunit is common to all gonadotropins, and the beta subunit is a specific subunit that is responsible for the biological activity of FSH [1]. FSH is secreted by gonadotropic cells of the anterior pituitary, and it stimulates the growth and recruitment of ovarian follicles in females and initiates spermatogenesis in males [2,3,4]. FSH is commonly used for the treatment of female infertility to induce ovulation in case of reversal anovulation or for ovarian hyperstimulation as a part of in vitro fertilization (IVF) [5]. However, the treatment of male infertility also requires the administration of FSH in some cases [6]. FSH binds to its receptor (FSHR) on the gonads and mediates signaling through the Gs/cAMP/protein kinase A pathway [6]. The receptor–ligand interface of the FSH–FSHR complex is formed mainly by amino acid residues of the beta-chain of FSH, but a free FSH beta-chain is not capable of binding the receptor with a sufficient affinity [7], and only a heterodimeric form of FSH is biologically active. FSH is a highly glycosylated molecule, and four N-linked carbohydrate moieties (two in the alpha subunit and two in the beta subunit) account for 30% of the protein mass and are present mainly in the form of highly branched tri- and tetra-antennary complex-type structures with a significant level of terminal sialylation [8]. Natural FSH in the bloodstream contains molecules with three N-linked glycans [9], and urine-derived FSH and recombinant FSH are completely N-glycosylated. More acidic (highly sialylated) glycoforms of FSH have a longer half-life in circulation due to decreased clearance by asialoglycoprotein receptors in the liver [10,11], but lower affinity to the FSH receptor [12]. FSH preparation with highly specific bioactivity should contain a mixture of slightly acidic and acidic forms [12]. The production of r-hFSH from Chinese hamster ovary (CHO) cells for medical use has been reported, and in recent years, other mammalian cells have been employed for the production of FSH (PERC.6 [13]). The urinary-derived FSH is still produced in large quantities, but r-hFSH is preferable because of its better batch-to-batch consistency, absence of human-derived infectious agents, and higher cost efficiency [14,15].

The original r-hFSH preparation Gonal-f^®^ (Merck Serono, Germany) has been in the market since 1995 [16], but of recent introduction to the market are three follitropin alpha biosimilars (Bemfola^®^, Gedeon Richter, Budapest, Hungary; Ovaleap^®^, Theramex, Dublin, Ireland; Primapur^®^, IVFarma, Moscow, Russia), which showed the same clinical efficacy and safety as original r-hFSH in randomized clinical trials [17]. The follitropin alpha biosimilar Primapur^®^ was developed, produced, and marketed in Russia in 2019 and has proven its efficacy and safety in pre-clinical studies [18], a phase I randomized crossover study (ClinicalTrials.gov Identifier: NCT03857230) for the safety and tolerability of a single subcutaneous dose [19], a phase III comparative randomized controlled study of therapeutic equivalence (NCT03088137) [20], and also in a retrospective, observational study (NCT04854707) comparing the efficacy of r-hFSH biosimilar therapy in real-world data of more than 5000 protocols of ovarian stimulation cycles [21,22].

Primapur^®^, comprising r-hFSH, was created using the cell line C-P1.3-FSH-G4 based on CHO cells, and it possesses a high specific productivity of 12.3 ± 1.7 pg/cell/day [23]. To isolate the protein from the conditioned culture medium, a proper downstream process is required. We have already reported a laboratory-scale downstream process based on immunoaffinity chromatography with highly specific single-domain recombinant camelid-derived antibodies [23]. The process is not similar to those usually employed for industrial purification of FSH, which involves several ultrafiltration/diafiltration operations [24]. The use of initial diafiltration for the clarified culture broth is usually mandated by low product concentration; however, in our case, the concentration of FSH was sufficient for economically feasible direct product capture by the multimodal chromatography resin. The immunoaffinity resin employed binds only the heterodimeric FSH in the alpha/beta chain interface; hence, both free FSH subunits, which are present in the culture broth in some quantities, are completely removed by the immunoaffinity step [25]. Immunoaffinity matrices with covalently bound single-domain recombinant camelid antibodies are expected to be significantly superior to conventional immunoaffinity matrices with immobilized murine monoclonal antibodies because of lower ligand leakage and complete absence of murine IgG in the eluate stream. Previously, we reported a laboratory-scale purification process for the B-domain-deleted blood clotting factor VIII with a single-domain antibody matrix. The resin used allowed the rapid and efficient removal of host cell-derived impurities and a reasonably high yield of the product [26]. The use of VIIISelect resin has also been described [27]. It has also been reported that single-domain antibody matrices are suitable for the purification of antibodies and antibody fragments [28]. In the case of FSH and the corresponding immunoaffinity resin, we found it to be suitable for the laboratory-scale purification process of FSH [23], but no data have been published to date on resin stability, practical dynamic binding capacity (DBC), and ligand leakage dynamics in repetitive use cycles.

The immunoaffinity purification of FSH enables the removal of both host cell-derived impurities and free FSH chains in one step; hence, the overall downstream process may be simplified to five column chromatography steps and two viral inactivation steps. This process, as partially described in [23], consists of an initial solvent/detergent viral inactivation step, performed on the clarified culture broth, multimodal, immunoaffinity, desalting by size exclusion, tandem ion exchange, and final size exclusion chromatography, viral filtration step, final potency adjustment, and sterile filtration.

In this paper, we present the results of the suitability studies of r-hFSH downstream process for the production of a pharmaceutical product with high biological activity and batch-to-batch consistency.

## 2. Materials and Methods

### 2.1. Materials

Ten r-hFSH samples used in this work are active pharmaceutical substances, representing a solution of biosimilar r-hFSH (IVFarma LLC, Moscow, Russia, batch nos. 0021019, 0031019, 0060820, 0070820, 0090920, 0101120, 0111120, 0121220, 0131220, 0010121). These samples were used to prepare the finished product Primapur^®^ in 2020–2021 at the contract manufacturing site (Zavod Medsintez LLC, Novouralsk, Russia). The chromatography resins used were Capto MMC (Cytiva, Marlborough, MA, USA), CaptureSelect FSH Affinity Matrix (Thermo Fisher Scientific, Waltham, MA, USA), Sephadex G-25 Medium (Cytiva), Capto Q (Cytiva), and Superdex 75 prep grade (pg, Cytiva). All other reagents used were of analytical grade and were purchased from commercial sources.

### 2.2. Overall Purification Process

**Figure 1:**
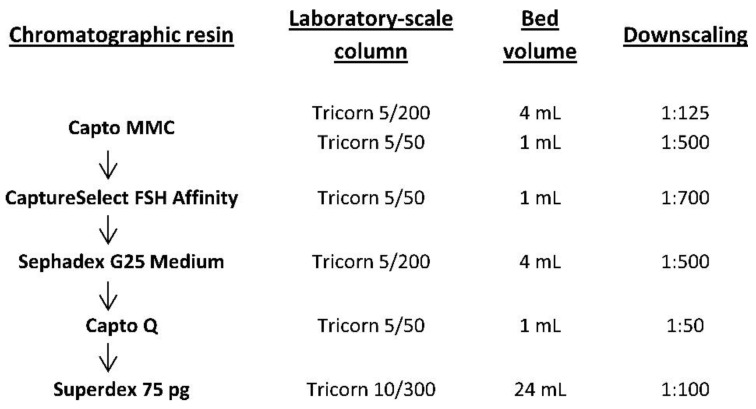
Overview of the downscaling of chromatographic purification steps in the production process of recombinant human follicle stimulating hormone (r-hFSH).

The overall downstream process for the isolation and purification of r-hFSH from culture medium includes the cell removal step (centrifugation/filtration), solvent/detergent viral inactivation step, five column chromatography steps (multimodal, immunoaffinity, desalting by size exclusion, tandem ion exchange, and final size exclusion chromatography), viral filtration step, formulation, and sterile filtration. Figure 1 represents the downscaling for laboratory-scale studies for all five chromatographic purification steps. To evaluate the degradation process of the resin in multiple purification cycles, cycling studies were performed. One chromatographic cycle, including column equilibration, sample loading, column wash, elution of the target protein, column cleaning-in-place (CIP), and sanitization-in-place (SIP), was repeated 40 times for each of the five chromatographic purification steps using a small downscaled column. Dynamic binding capacity (DBC) was also determined for multimodal, immunoaffinity, and ion-exchange chromatography.

### 2.3. Cell Cultivation, Harvesting, and Viral Inactivation

r-hFSH for downstream process studies was obtained by the proprietary extended batch culturing process in the wave-mixed bioreactor Biostat RM (Sartorius, Gottingen, Germany). The harvested culture medium from the bioreactor was clarified by centrifugation at 600× *g* for 5 min and 20,000× *g* for 15 min, after which a solvent/detergent viral inactivation step was performed by adding tri-n-butyl phosphate to 0.3% and Tween 80 to 1% (*v/v*). After 12 h incubation at room temperature, the pH was adjusted to 5.5 by adding hydrochloric acid, and the medium was centrifuged at 20,000× *g* for 15 min.

### 2.4. Multimodal Resin Chromatography (Step 1)

The initial product capture step aimed at isolation and concentration was performed using two columns filled with Capto MMC resin: a Tricorn 5/200 column (Cytiva) packed with 4 mL of the resin was used for DBC determination (downscaling 1:125, column volume/column volume, CV/CV), and a Tricorn 5/50 column (Cytiva) packed with 1 mL of the resin was used to determine the optimal residence time and the cycling studies (downscaling 1:500, CV/CV). The column was equilibrated with 20 CV of 50 mM Na-PO_4_ (pH 5.5), 3 mM methionine, 100 mM NaCl solution at a flow rate of 2 mL/min. The sample (filtered harvested medium after the viral inactivation step) was applied at a flow rate of 1 mL/min, and the wash step was performed with 40 CV of the same solution. Elution was performed using 15 CV of 50 mM Tris-HCl (pH 7.5), 3 mM methionine, 150 mM NaCl solution at a flow rate of 1 mL/min, in the reverse flow direction. The column CIP procedure was performed in reverse flow direction with 20 CV of 0.5 M NaOH, 20% ethanol, 0.1% Triton X-100 at a flow rate of 2 mL/min, followed by the final application of 20% ethanol SIP/storage solution (20 CV).

To determine the DBC, the clarified culture medium containing r-hFSH at a concentration of 24 mg/L was loaded on the Tricorn 5/200 column (bed volume 4 mL) with the Capto MMC resin at a flow rate of 2 mL/min (residence time 2 min). The flow-through fractions during the column load were collected, and the non-bound FSH was measured by enzyme-linked immunosorbent assay (ELISA), as previously described. Percentage breakthrough (calculated by dividing the concentration of FSH in the flow through by the concentration of FSH in the loaded sample) was plotted against the amount of protein loaded to generate the breakthrough curve. From the breakthrough curve, the DBC at 10% breakthrough (DBC_10%_) was determined as the amount of FSH loaded on 1 mL of packed resin until the FSH concentration in the flowthrough reached 10% of the concentration in the applied sample.

To determine the optimal residence time, the same sample was applied to the Tricorn 5/50 column (bed volume 1 mL) at various flow rates, namely 1.5 mL/min (residence time 40 s) and 1 mL/min (residence time 1 min), and breakthrough curves were generated and target protein loss at DBC_10%_ was calculated as the amount of FSH in the flowthrough (area under breakthrough curve) divided by the total amount of applied FSH. In the cycling studies; the described purification cycle was conducted 40 times using a Tricorn 5/50 column (bed volume 1 mL). The volume of the eluate peak was calculated for each cycle by peak integration using Unicorn 5.11 software (Cytiva).

### 2.5. Immunoaffinity Chromatography (Step 2)

The immunoaffinity chromatography step for selective purification of the intact r-hFSH molecule was downscaled to 1:700 (CV/CV). A Tricorn 5/50 column packed with 1 mL of CaptureSelect FSH Affinity Matrix was used for the process studies. The column was equilibrated with 10 CV of 20 mM Tris-HCl (pH 7.5), 3 mM methionine, 150 mM NaCl solution at a flow rate of 0.5 mL/min. The eluate from step 1 was applied at a flow rate of 0.2 mL/min, and the wash step was performed with 10 CV of the same solution. Elution was performed with 5 CV of 2 M MgCl_2_, 50 mM Tris-HCl (pH 7.5), and 3 mM methionine solution at a flow rate of 0.2 mL/min, in the reverse flow direction. The column CIP procedure was performed in the reverse flow direction with 10 CV of 100 mM glycine-HCl (pH 2.0) at a flow rate of 1 mL/min, followed by the final application of 20% ethanol SIP/storage solution (10 CV). Forty cycles were performed, and the eluate peak volumes were determined as earlier described.

### 2.6. Desalting by Size Exclusion (Step 3)

Desalting the eluate from step 2 for further ion-exchange chromatography was performed by size exclusion at a 1:500 downscale (CV/CV). A Tricorn 5/200 column packed with 4 mL of Sephadex G25 Medium resin was used for the process studies. The column was equilibrated with 2.5 CV of 20 mM Tris-HCl (pH 7.0) solution at a flow rate of 1 mL/min. The model solution (0.5 mg/mL BSA, 2 M MgCl_2_) was applied in portions (0.75 mL at a flow rate of 1 mL/min), and elution was performed with 1.8 CV of the same buffer at a flow rate of 1 mL/min. Six consecutive loads were applied before the cleaning. The column CIP procedure was performed with 3.5 CV of 0.5 M NaOH at a flow rate of 1 mL/min, followed by the application of 20% ethanol SIP/storage solution (4 CV). To assess the optimal load volume, various sample volumes (0.5–1.25 mL) were applied on the column at a constant volumetric flow rate of 0.5 mL/min, and a change in resolution between the elution peaks of BSA and MgCl_2_ in the channels of absorbance at 280 nm, and conductivity was monitored.

To estimate the optimal flow rate, the same load volume (0.75 mL) was applied at various flow rates at a range of 0.4–1.4 mL/min, and a change in resolution was monitored.

In the cycling studies, the described desalting cycle was conducted 40 times (resulting in 240 column loads) on the same filled column, and the width at half height of the eluate peak was calculated for each cycle using Unicorn 5.11 software.

### 2.7. Tandem Ion Exchange Chromatography (Step 4)

The separation of differently charged FSH variants is performed in the manufacturing process by combining a cation exchange membrane adsorber Sartobind S (Sartorius) and anion exchange column with Capto Q resin. This step was downscaled to 1:50 (CV/CV). The Sartobind S membrane was not tested for stability because it is a disposable device, but the step involving membrane wash prior to use was included in each test cycle. A Tricorn 5/50 column packed with 1 mL of Capto Q resin was used for the process studies. Because of the very high loading capacity of Capto Q resin, even the smallest Tricorn 5/50 column (CV 1 mL) was expected to adsorb more than 30 mg of the FSH at once; hence, the resin degradation study on the real product was expected to involve several grams of the semi-purified FSH. BSA was used as a model protein for the resin degradation study (10 mg/mL BSA, 20 mM Tris-HCl (pH 7.0), and 3 mM methionine). In the control cycles (every 10th cycle), excess BSA was applied to the column to assess DBC change. The column was equilibrated with 5 CV of 20 mM Tris-HCl (pH 7.0) and 3 mM methionine solution at a flow rate of 1 mL/min. The column was washed with 5 CV of 0.5 M NaOH and 2 M NaCl buffer at a flow rate of 0.2 mL/min, and neutralized by 6 CV of 20 mM Tris-HCl (pH 7.0), 3 mM methionine, and 25 mM NaCl solution at a flow rate of 1 mL/min. This step represented the initial wash and neutralization of the S-membrane adsorber. The column was re-equilibrated with 3 CV of the same equilibration buffer, and the sample (0.8 mg/mL FSH solution in control cycles, 10 mg/mL BSA solution in working cycles) was applied at a flow rate of 0.6 mL/min. Wash steps were performed at a flow rate of 1 mL/min with the following solutions: 4 CV of equilibration solution, 6 CV of 20 mM Tris-HCl (pH 8.5), 3 mM methionine solution (wash 1), 10 CV of 20 mM Tris-HCl (pH 8.5), 3 mM methionine, 80 mM NaCl solution (wash 2), and 6 CV of wash 1 solution. The target FSH fraction was eluted with 5 CV of 20 mM Tris-HCl (pH 8.5), 3 mM methionine, and 250 mM NaCl solution at a flow rate of 0.6 mL/min in the reverse flow direction. The column CIP procedure was performed in the reverse flow direction with 1 M NaOH solution at a flow rate of 1 mL/min (4 CV) and 0.08 mL/min (5 CV) and total CIP time 1 h, followed by the application of 20% ethanol SIP/storage solution (10 CV). To determine the DBC for FSH, 0.8 mg/mL FSH, 20 mM Tris-HCl (pH 7.0), and 3 mM methionine solution was applied on the column in cycle 22. The DBC was calculated as earlier described.

In the cycling studies, the described purification cycle was conducted 40 times, and eluate peak integration was performed using Unicorn 5.11 software.

### 2.8. Final Size Exclusion Purification (Step 5)

The final purification step, which involved the use of size-exclusion column and aimed at clearance from protein aggregates, free subunits, and low molecular weight contaminants, was performed at a 1:100 downscale (CV/CV). A Tricorn 10/300 column packed with 24 mL of Superdex75 pg resin was used for the process studies. The column was equilibrated with 2 CV of 10 mM Na-PO_4_ (pH 7.2) and 3 mM methionine solution at a flow rate of 1 mL/min. The model solution (10 mg/mL BSA, 20 mM Tris-HCl (pH 8.5), 3 mM methionine, and 250 mM NaCl) was applied at a flow rate of 0.3 mL/min. The sample volume was 1 mL, and elution was performed with 1.2 CV of the same buffer at a flow rate of 0.3 mL/min. The column CIP procedure was performed using 0.5 M NaOH solution at a flow rate of 1 mL/min (1.2 CV) and 0.13 mL/min (0.33 CV), and the CIP solution was neutralized with 1.2 CV (0.2 M CH_3_COOH, 0.2 M CH_3_COONa solution) at a flow rate of 1 mL/min, followed by the application of 20% ethanol SIP/storage solution (2 CV). The described purification cycle was conducted 40 times, and the width at half height of the eluate peak was calculated for each cycle using Unicorn 5.11 software.

We estimated the elution volume generated in purification step 4 (ion exchange chromatography) by typical column loading and applied this sample volume to the final size-exclusion column in this step.

### 2.9. Viral Clearance Studies

A part of the work involving the cultivation and preparation of virus was carried out in the Laboratory of the Russian State Collection of Viruses, D. I. Ivanovskiy Institute of Virology, Division of N.F. Gamaleya National Research Center of Epidemiology and Microbiology of the Ministry of Health of the Russian Federation. The work was carried out in full compliance with the legislative international and national acts regulating activities related to pathogenic microorganisms of groups II–IV pathogenicity. Viral clearance studies were performed to demonstrate the suitability of the process to eliminate the following model viruses: pseudorabies virus (PRV), parainfluenza virus (PIV), reovirus type-3 (REO), and encephalomyocarditis virus (EMC). According to a previous study [29], CHO cells and, specifically, master and working cell banks of the cell line C-P1.3-FSH-G4 are related to “Case A” when studies with non-specific model viruses are appropriate. The r-hFSH-containing culture medium from CHO cell line was spiked with the mentioned viruses at an activity of not less than 7.0 lg TCD_50_/mL, and viral inactivation, multimodal chromatography, immunoaffinity chromatography, and ion-exchange chromatography steps were conducted as earlier described. The final nanofiltration step was performed using a Virosart CPV Capsule (Sartorius). After each investigated step of chromatographic purification, the cytotoxic properties of the solutions were tested using three cell lines: MDBK, BHK-21, and L929 (Russian collection of cell lines, Saint-Petersburg, Russia). Reduction factor (RF) was evaluated at every step to demonstrate the downstream removal of virus.

### 2.10. Quantification of FSH by ELISA

r-hFSH was quantified in culture medium or in semi-purified chromatographic fractions using a DRG FSH ELISA kit (DRG, Germany) according to the manufacturer’s guidelines. The samples were diluted with 1% bovine serum albumin (BSA) in Tris-buffered saline (TBS). To calculate the concentration in mass units, a conversion factor of 1 IU of r-hFSH = 0.083 μg was used.

### 2.11. Analysis of Leachables from CaptureSelect FSH Affinity Matrix

CaptureSelect FSH Affinity Matrix resin leakage was evaluated by ELISA using a CaptureSelect FSH Ligand Leakage kit (Life Technologies, Carlsbad, CA, USA). MgCl_2_ at a high concentration can affect antibody interaction; hence, the eluates from the affinity column were subjected to diafiltration in exchange with TBS solution using a Vivaspin 500 concentrator (Sartorius, Goettingen, Germany) with a 3 kDa molecular weight cut-off to retain the affinity ligand, which is a 14 kDa recombinant mini-antibody. The final concentration of MgCl_2_ in the samples was less than 16 mM. The samples were diluted with 1% BSA-TBS. ELISA was performed according to the manufacturer’s guidelines, with the use of placebo solution as diluent for the analysis of r-hFSH drug substance.

### 2.12. Analysis of Residual Host Cell Proteins (HCP)

The concentration of residual CHO proteins in the eluates from the affinity column was measured using ELISA with a CHO-CM HCP ELISA Kit CM015 (Cygnus, Dorchester, UK). The same sample preparation was performed as described for the analysis of leachables from CaptureSelect FSH Affinity Matrix, and the desalted samples were diluted with 1% BSA-TBS. ELISA was performed in accordance with the manufacturer’s guidelines. The concentration of residual HCP in the r-hFSH drug substance batches was measured in the same way, except for the use of formulation solution (10 mM Na-PO_4_, 5 g/L L-methionine, 68.46 g/L sucrose, 0.02 g/L Tween-20, pH 7.0) as diluent for the generation of the calibration curve. The test r-hFSH substance was used without sample preparation.

### 2.13. Analysis of Residual Host-Cell DNA

The residual CHO DNA in the drug substance was determined by quantitative real-time polymerase chain reaction (qPCR) using Luminaris qPCR Master Mix (Thermo Fisher Scientific). The primers and the probe were as follows: CHO49F (5′-TGGAGAGATGGCTCGAGGTT-3′) and CHO49R (5′-TGGTTGCTGGGAATTGAACTC-3′) at a final concentration of 0.4 μM each as well as CHO49pr (5′-FAM-AGAGCACCAACTGCTCTTCCAGAGGTCC-BHQ1-3′) at a final concentration of 0.27 μM. Real-time PCR was performed using a DTlite PCR System (DNA-Technology, Moscow, Russia) under the following cycling conditions: initial denaturation at 95 °C, 45 cycles of denaturation at 95 °C, annealing at 49 °C, and extension at 72 °C. The r-hFSH drug substance used for analysis was used without sample preparation.

### 2.14. Analysis of Bound Sugars in r-hFSH

Analysis of the carbohydrate moieties attached to the FSH molecule (bound sugars) was performed using the colorimetric method previously described by the authors of [30] with some modifications. To eliminate sucrose, which is present in the formulation, the r-hFSH drug substance was subjected to diafiltration in exchange with TBS solution using a Vivaspin 500 concentrator (Sartorius) with a molecular weight cut-off of 10 kDa. The residual sucrose concentration was below the quantification limit. Glucose solutions were used to generate a standard curve. Phenol was added to the desalted FSH solution and the standard samples to a final concentration of 0.6%; concentrated sulfuric acid (two-third volume) was added rapidly, and the tube was vortexed. After incubation for 5 min at 95 °C on ice, the color developed, and absorption at 490 nm was measured.

### 2.15. In Vivo r-hFSH Activity Assay

r-hFSH biological activity was determined according to the European Pharmacopoeia (Chap. 2286), as earlier described [22], at the GMP/GLP compiled laboratory Bioassay GmbH (Heidelberg, Germany). The in vivo biological activity of follitropin alpha biosimilar was 13.6 IU/μg, and according to European Pharmacopoeia, this stated activity should be not less than 10.9 IU/μg (or 80% of stated activity) and not more than 17.0 IU/μg (125%), 95% confidence interval: 8.7 IU/μg (64%)–21.1 IU/μg (156%).

### 2.16. N-Glycan Analysis

N-glycan profile was determined according to the European Pharmacopoeia (Chap. 2286) with some modifications. A chromatographic separation was performed using GlycanPac™ AXH-1 HPLC column AXH-1 (cat# 082470, Thermo Fisher Scientific). The resulting Z-index was calculated as the area of each determined peak for neutral, mono-, di-, tri-, and tetra-sialylated forms of r-hFSH sample, compared with Follitropin CRS for peptide mapping and glycan analysis (European Pharmacopoeia Reference Standard, cat no. Y0001627). The Z-index was calculated using the following expression: (A0 × 0) + (A1 × 1) + (A2 × 2) + (A3 × 3) + (A4 × 4), where A0 = peak area percentage due to the neutral form; A1 = peak area percentage due to the mono-sialylated form; A2 = peak area percentage due to the di-sialylated form; A3 = peak area percentage due to the tri-sialylated form; and A4 = peak area percentage due to the tetra-sialylated form. The Z number obtained for the test and reference solutions of r-hFSH was in the range of 177–233, according to the follitropin monograph of the European Pharmacopoeia.

### 2.17. Analytical Size-Exclusion Chromatography

The levels of r-hFSH content (0.4–0.8 mg/mL), free subunits (maximum 3%), and oligomers (maximum 0.5%) were estimated by size-exclusion chromatography according to the European Pharmacopoeia (Chap. 2286), with some modifications. An internal reference standard for r-hFSH was developed, which contained approximately 10% free r-hFSH subunits and 10% r-hFSH oligomers. The system suitability was accepted when the chromatogram of the internal reference standard of r-hFSH had a mean resolution between peaks of oligomer and r-hFSH heterodimer ≥ 1 and between r-hFSH heterodimer and free subunits not less than 1.1.

### 2.18. Analytical Reverse Phase Chromatography

To determine the oxidized r-hFSH forms (maximum 6%), reverse-phase chromatography was performed according to the European Pharmacopoeia (Chap. 2286).

### 2.19. Statistical Analysis

*T*-tests were performed using Prism (GraphPad Software, San Diego, CA, USA) available at https://www.graphpad.com/quickcalcs/ttest1.cfm (accessed on 22 November 2021).

## 3. Results

Downstream processing of biopharmaceuticals may significantly affect their cost, composition of charge variants, and levels of impurities, and it may consequently change the quality of the drug itself. We present the results of process studies for the FSH biosimilar drug, manufactured by a significantly simplified downstream process devoid of tangential flow filtration operations. The study involved the estimation of DBC, optimal residence time, and possible number of cycles for each chromatographic step, that is, the evaluation of the resin capability to maintain its chromatographic properties without loss in binding efficiency. Scale equivalence for the steps related to size exclusion was achieved by maintaining bed height and linear flow velocity similar to that used in the industrial production of r-hFSH while varying the column diameter. DBC and residence time were studied using shorter bed heights to save sample and overall column operation time.

### 3.1. Multimodal Chromatography Using Capto MMC Resin (Step 1)

This step was conducted using a small-scale column size (at a scale of 1:125–1:400). A Tricorn 5/200 column with a bed height of 20 cm and a packed bed volume of 4 mL, which corresponded to the bed height of the industrial column, was used to evaluate DBC and residence time. The linear flow rates of eluents in these experiments for short columns were limited to 200–600 cm/h. This is consistent with the recommendation of the resin manufacturer for a bed height of 20 cm, which is usually used for industrial production.

To determine DBC, we applied to the column a clarified culture medium after viral inactivation, with 24 mg/L FSH, and quantified the non-binding FSH in the flowthrough using ELISA. The FSH content in the non-binding fraction took the form of an S-shaped curve. At a residence time of 2 min, FSH started to appear in flowthrough and reached a concentration of 10% from that in the applied medium after loading of 75 CV of culture medium or 7.4 mg FSH, which corresponded to 1.9 mg FSH/mL resin (Figure S1A). Therefore, the DBC at 10% breakthrough (DBC_10%_) was set as 1.9 mg FSH/mL of Capto MMC resin. The loading density was specified as 70% of DBC_10%_ and set as 1.3 mg FSH/mL of Capto MMC resin. It should be noted that the target protein was bound to the resin along with many other CHO proteins, and the FSH content in the Capto MMC eluate was only 28% for the experimental sample of the culture medium. The apparent low DBC of Capto MMC resin toward FSH corresponded to more than 10 mg/mL capacity for all proteins bound.

For the typical FSH concentration of 40 mg/L in the culture medium, direct application of the virus-inactivated medium to a Capto MMC column up to the estimated loading capacity at 2 min residence time will take more than 100 min. To shorten the expected sample application time, we evaluated the DBC at shorter residence times (1 min and 40 s) using a smaller 5/50 Tricorn column with a bed volume of 1 mL.

**Figure 2:**
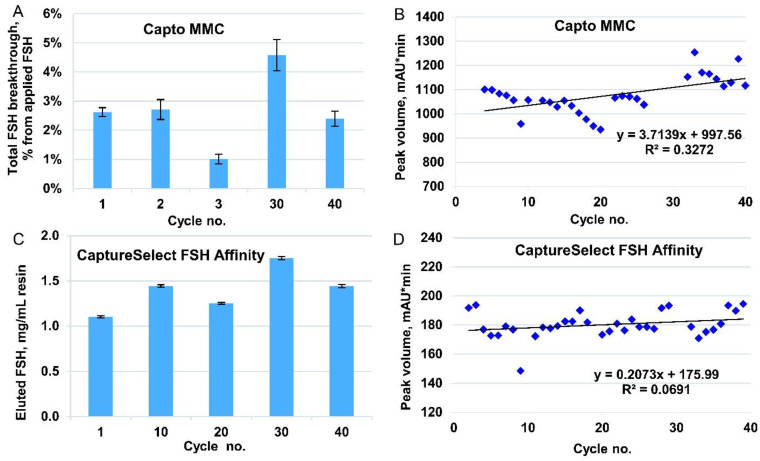
Stability studies for multimodal chromatography (step 1) and immunoaffinity chromatography (step 2). (**A**) Total FSH breakthrough at applying 1.9 mg FSH/mL Capto MMC resin in control cycles (area under breakthrough curve) as measured by enzyme-linked immunosorbent assay. Downscaling 1:125 in cycle 1 (Tricorn 5/200 column, bed volume 4 mL), 1:500 in the remaining cycles (Tricorn 5/50 column, bed volume 1 mL). Loaded 1.9 mg FSH/mL resin, residence time 2 min (cycle 1), 40 s (cycle 2), 1 min (cycles 3, 30, 40). Data are mean values, and error bars represent standard deviation (n = 2). (**B**) Peak volume integration for eluates in 40 working cycles, absorbance channel at 280 nm. Capto MMC resin, downscaling 1:400 (Tricorn 5/50 column, bed volume 1 mL). Loaded 1.3 mg FSH/mL resin, residence time 1 min. (**C**) FSH eluted from immunoaffinity column. CaptureSelect FSH Affinity Matrix, downscaling 1:700 (Tricorn 5/50 column, bed volume 1 mL). Loaded 2 mg FSH/mL resin, residence time 1 min. FSH content in the eluates measured by absorption at 280 nm. (**D**) Peak volume integration for eluates from 1 mL column filled with CaptureSelect FSH Affinity Matrix. Loaded 1.1 mg FSH/mL resin, downscaling 1:700, residence time 1 min. Data for 40 working cycles in absorbance channel at 280 nm.

As shown in Figure 2A and Table 1, no significant decrease in DBC was observed at various residence times. The loss of the target protein (ratio of FSH quantities in the sample applied and in the flow-through fraction) did not exceed 3% in all the three cases, and it was the lowest at a residence time of 1 min. Therefore, we set the optimal residence time to 1 min, which corresponded to the recommended maximal linear flow rate for production chromatography columns for Capto MMC resin and the expected sample application time of 50 min.

Cycling studies were conducted for a 1 mL column. To assess the degradation of resin, the cycle of equilibration, application, washing, elution, regeneration, and sanitization was repeated 40 times. After every 10 successive cycles, DBC was determined (Figure 2A), and for cycles 3, 30, and 40, the dynamics of FSH content in the flow-through fractions were also monitored (Figure S1B). Elution peak volumes were determined by peak integration for 3–40 cycles, and no significant decrease in binding capacity was observed (Figure 2B).

### 3.2. Immunoaffinity Chromatography Using CaptureSelect FSH Affinity Matrix (Step 2)

First, we determined the static binding capacity for the CaptureSelect FSH Affinity resin, which was 3 mg FSH/mL resin, that is, the maximal load after which the concentration of the target protein in the non-binding fraction was the same as that in the applied medium. To determine the DBC, we applied to the 1 mL column pooled eluate from step 1 with 0.1 mg/mL FSH and fractionated the flow-through sample; the load was 2 mg/mL FSH in control cycles 1, 10, 20, 30, 40, and 1 mg/mL in all other cycles (the working cycles). The DBC_10%_ in cycle 1 was 1.6 mg/mL (Figure S2) and remained the same during the next 30 cycles of use. In cycle 40, we noticed some loss in binding capacity after achieving 10% breakthrough by applying approximately 1.2 mg FSH/mL (Figure S2). Therefore, for every cycle, we also monitored the FSH eluted from the column by absorption at 280 nm (Figure 2C for control cycles and Figure 2D for working cycles). All the elution data showed that no significant deterioration in binding capability appeared during 40 performance cycles.

Because the purpose of this stage of purification was to obtain the maximum amount of pure product at the exit from the column, the loading density was specified as 90% of the DBC_10%_ at cycle 40, that is, 1.1 mg FSH/mL resin, which is consistent with the data on the amount of eluted FSH from the column. We tested only a residence time of 5 min without further variations, as this residence time corresponds to the maximum linear flow rate recommended by the manufacturer of the resin for industrial-scale columns.

**Figure 3:**
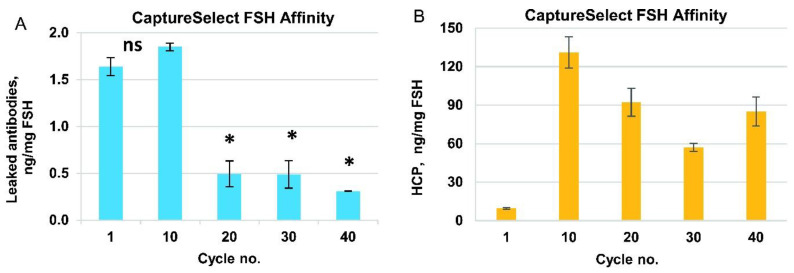
Purity characterization of the immunoaffinity column eluate (CaptureSelect FSH Affinity Matrix). Data for control cycles. (**A**) Camelid anti-FSH mini-antibodies concentration as measured by enzyme-linked immunosorbent assay (ELISA). (**B**) Residual host-cell proteins (HCP) concentration as measured by ELISA. All data are mean, error bars represent standard deviation (*n* = 2). *—*p*-value < 0.05, ns—*p*-value > 0.05, unpaired *t*-test.

To confirm the efficiency of HCP removal by this purification step, we measured the HCP content in the eluates for control cycles 1, 10, 20, 30, and 40 using ELISA. The range of HCP content in the FSH was 10–130 ng/mg (Figure 3B), which was close to the typical limit of the biopharmaceutical drug substance (20 ng/mg). We observed that the HCP level in the first use cycle was very low, and for cycles 10–40, it was much higher and approximately the same (60–120 ng/mg). We propose that the majority of the HCP in the product stream arose from the protein–protein complexes formed around the host cell proteins, covalently bound to the resin during first-use cycles. No specific resin inactivation by the primary amines was performed before use with the semi-purified FSH solution in order to not intentionally covalently bind to any charged groups in the column. The eluate of the Capto MMC column contained approximately 72% of HCP (all proteins other than FSH are considered as HCP), and the purification degree of the CaptureSelect FSH Affinity resin was estimated as 30,000-fold, which was significantly higher than that of the typical immunoaffinity column.

Leaked anti-FSH mini-antibodies were also analyzed for the control cycles 1, 10, 20, 30, and 40 by performing ELISA using a CaptureSelect FSH Ligand Leakage kit. The concentration of anti-FSH antibodies was about 2 ng/mg FSH in the first 10 cycles, then dropped to approximately 0.5 ng/mg for cycle 20 and remained at this level for the next 30 cycles (Figure 3A). This level of the leaked mini-antibody (0.5–2 ppm) was relatively low but slightly exceeded the limit of quantification for the ELISA detection method. At the same time, for three actual batches of FSH, produced by the downstream process developed, the measured leaked mini-antibody admixture level was below the quantification limit of the assay and close to the limit of detection in the 100–130 ppb range (Table 2). The degree of removal of leaked mini-antibodies by the overall purification process may be estimated to be at least 20-fold.

### 3.3. Desalting Using Sephadex G25 Medium Resin (Step 3)

To study the desalting step, we used a column with a bed height similar to that used in the production process (20 cm) and a minimal practical diameter of 5 mm (CV 4 mL). This process removes excess MgCl_2_ from the product stream, and the composition of the macromolecular components of the FSH solution is not expected to change. We used an imitator solution of BSA (0.5 mg/mL BSA in 2 M MgCl_2_ instead of FSH) to limit the consumption of valuable protein. This process consumes a large volume of desalting solution and takes a relatively long time; hence, the optimization of both column load and flow was performed before the resin degradation study. First, we applied different sample volumes (0.5–1.25 mL) at a constant volumetric flow rate of 0.5 mL/min and observed a change in resolution between the elution peaks of BSA and MgCl_2_, determined at optical density of 280 nm and conductivity (Figure S3A). Based on the data obtained, we set the optimal load volume as 0.75 mL (19% of the CV), where peak overlapping was 1.4%. This level of peak overlapping, that is, this level of residual MgCl_2_ in the product elution fraction, should be approximately 7 mM, far below the salt concentration, which may affect subsequent ion-exchange chromatography operations.

To estimate the optimal flow rate, we applied a sample volume of 0.75 mL at various flow rates between 0.4 and 1.4 mL/min and observed that the best overall resolution between the peaks in the optical density and conductivity channels lies in the range of 0.9 and 1.4 mL/min flow; however, at 1.4 mL/min, the degree of overlapping of the peaks increased dramatically (Figure S3B). Thus, we set the optimal flow rate for the application and elution of sample at 1 mL/min (300 cm/h).

The criterion for stopping the eluate collection was set as the increase in conductivity from 1.3 mS (the conductivity of the equilibration buffer solution) to 2.0 mS, which corresponded to 0.6% of MgCl_2_ in the sample applied. The overall MgCl_2_ concentration in the eluate in this case was approximately 1 mM, whereas the concentration of chloride anions in the running buffer for step 3 (Tris-HCl 20 mM) was approximately 20 mM.

**Figure 4:**
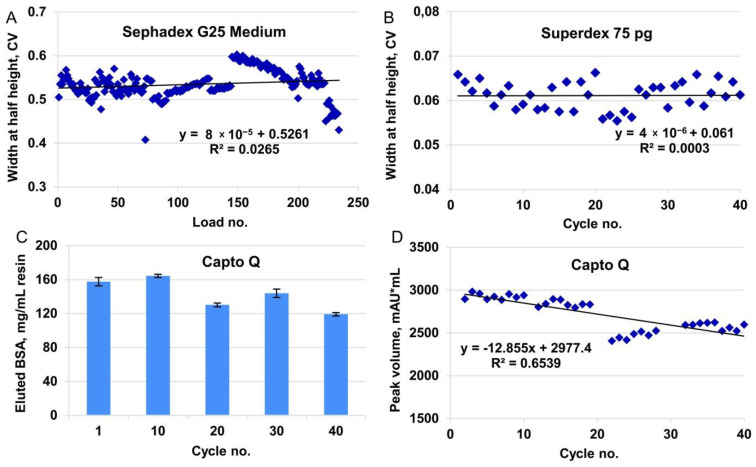
Stability studies for desalting by size-exclusion, ion exchange chromatography, and final size-exclusion chromatography (steps 3, 4, and 5, accordingly). (**A**) Eluate peak broadening for Tricorn 5/200 column, bed volume 4 mL, Sephadex G25 Medium resin (downscaling 1:500), conductivity channel, 40 cycles, six loads in each cycle. Sample volume 0.75 mL. (**B**) Eluate peak broadening for Tricorn 10/300 column, bed volume 24 mL, Superdex75 pg resin (downscaling 1:100), absorbance channel at 280 nm, 40 cycles. Sample volume 1 mL. (**C**) BSA eluted from Capto Q column in control cycles. Tricorn 5/50 column, bed volume 1 mL, Capto Q resin (downscaling 1:50). Loaded 320 mg BSA/mL resin, residence time 1.67 min. BSA content measured by absorption at 280 nm of the collected eluates. (**D**) Peak volume integration for eluates in working cycles, absorbance channel at 280 nm. Same column as in panel C. Loaded 30 mg BSA/mL resin, residence time 1.67 min.

To test the behavior of the resin in multiple cycles, we successively applied six portions of the sample under the established conditions (load volume 0.75 mL, flow rate, 1 mL/min), followed by regeneration (CIP) and sanitization, which was repeated for 40 cycles. No peak broadening was observed during 240 successive sample applications (Figure 4A).

### 3.4. Ion-Exchange Chromatography Using Capto Q Resin (Step 4)

The polishing of FSH from the leaked mini-antibodies and the rest of the HCP was performed by using the Sartobind S membrane adsorber connected to the Capto Q column. The anion-exchange column was expected to bind FSH, but not some HCP, and allow the elution of FSH before that of the residual host-cell DNA, process-derived endotoxins, and some HCP. Its main purpose is the selective elution of neutral FSH forms and subsequent elution of the highly concentrated pure FSH solution directly to the size-exclusion column for the final purification and removal of the non-excipient salts. The Sartobind S membrane used in the manufacturing process was not used in these process studies due to its disposability; however, the step involving the membrane wash was included in each column use cycle. Because of the very high loading capacity of Capto Q resin, even the smallest Tricorn 5/50 column (column volume 1 mL) was expected to adsorb more than 30 mg of the FSH at once; hence, the resin degradation study utilizing the real product was expected to consume several grams of semi-purified FSH. BSA was used as a model protein for the resin degradation study (10 mg/mL BSA, 20 mM Tris-HCl pH 7.0, 3 mM methionine). To determine the DBC of the FSH for Capto Q resin, we used a 0.8 mg/mL FSH, 20 mM Tris-HCl (pH 7.0), and 3 mM methionine solution, closely resembling the actual desalted affinity column eluate. We applied 80 mg of FSH on a 1 mL Capto Q column, fractionated the flow-through samples, and measured the protein content in the collected fractions by absorption at 280 nm. The static binding capacity of the Capto Q resin toward FSH was not achieved, and the DBC_10%_ of FSH at a residence time of 1 min was determined to be 70 mg FSH/mL resin (Figure S4A).

For the cycling studies, using a model solution, the sample load of BSA in working cycles was 30 mg/mL, which was the same as the typical process mass load of FSH; for control cycles, the BSA load was increased to 320 mg/mL, which corresponded to the static binding capacity found for the model protein. The DBC_10%_ of BSA for Capto Q resin was 200 mg/mL resin at 1 min residence time. The change in DBC was monitored by two parameters: absorption curve during application of BSA on the column (BSA dynamics in flow through, Figure S4B), and quantity of the eluted BSA (measured by the UV absorption of the collected eluate for control cycles, Figure 4C, and by integration of peak volume in working cycles, Figure 4D).

According to the data for both elution and breakthrough monitoring, some deterioration in the resin binding capacity was observed in the late cycles. However, such deterioration did not affect the production process because the typical FSH load on the production scale column was 30 mg/mL, which was twice as small as DBC_10%_ of FSH in this study.

### 3.5. Final Size Exclusion Chromatography Using Superdex75 pg Resin (Step 5)

To study the final purification step, we used a column with a bed height equal to that used in the production process (30 cm) and a smaller diameter (Tricorn 10/300 filled with 24 mL of Superdex 75 pg resin). Imitator solution of 10 mg/mL BSA, 20 mM Tris-HCl (pH 8.5), 3 mM methionine, and 250 mM NaCl was used for resin degradation studies. The FSH solution was omitted because of the practical absence of the multimer fraction in the FSH eluted from the Capto Q resin.

To establish the sample volume to be loaded on the size-exclusion column, we first estimated the elution volume generated in purification step 4 (ion exchange chromatography) and observed that the typically loaded amount of FSH was eluted within 2 CV (Figure S4C), which was approximately 4% of the column volume of step 5. This ratio of the sample volume and the packed column volume fit the range recommended by the resin manufacturer (0.5–4%). The cycling studies were carried out at a scale of 1:100, and hence, a sample volume of 1 mL was applied to the 24 mL column. No peak broadening was observed during the 40 full-column use cycles (Figure 4B).

### 3.6. Viral Clearance Studies

Viral clearance studies were performed to demonstrate the suitability of the process to eliminate the following model viruses: PRV, PIV, REO, and EMC. The RF for the model viruses—PRV, PIV, REO, and EMCV (Table 3)—exceeded 12 log_10_ for each virus, indicating a high degree of viral elimination during the downstream process (Table 3).

The measured concentration of viruses in cell culture harvest was below the sensitivity of transmission electron microscope resolution (1.0 × 10^3^ viral particles/mL), which was assumed as the theoretical maximum concentration of virus in cell culture harvest. The maximum daily dose of r-hFSH is 450 IU, which is equal to 33 mg of r-hFSH, and the volume of harvest needed to make the required dose is 1.1 mL. The estimated particles per dose can be calculated according to the following equation [29]:

$$\frac{\left(\mathrm{Virus}\;\mathrm{units}\;\mathrm{in}\;\mathrm{harvest}\;\mathrm{per}\;\mathrm{mL}\right)*\left(\mathrm{mL}\;\mathrm{of}\;\mathrm{harvest}\;\mathrm{to}\;\mathrm{make}\;a\;\mathrm{dose}\;\mathrm{of}\;r-\mathrm{hFSH}\right)}{\mathrm{Total}\;\mathrm{clearence}\;\mathrm{factor}\;\mathrm{for}\;\mathrm{each}\;\mathrm{model}\;\mathrm{virus}\;\left(\mathrm{from}\;\mathrm{Table}\;3\right),}$$

results represented in Table 4.

Therefore, the developed purification process of r-hFSH successfully removed the model viral particles, providing a high degree of safety for the finished medicinal product.

### 3.7. Batch-to-Batch Consistency

**Figure 5:**
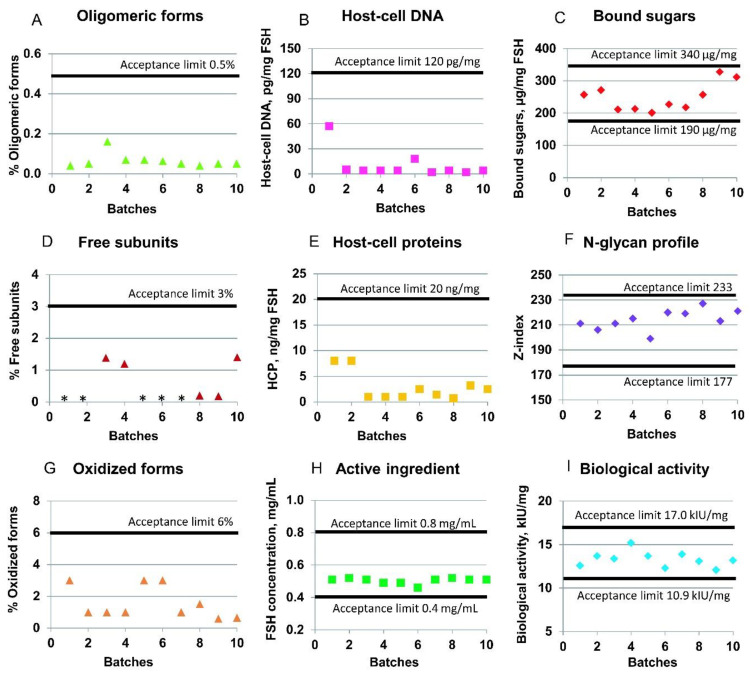
Quality control parameters of the r-hFSH drug substance. (**A**) Oligomeric forms content; (**D**) free subunit content; (**G**) oxidized forms content; (**B**) host-cell DNA content; (**E**) host-cell proteins (HCP) content; (**H**) level of the active ingredient; (**C**) bound sugars in the r-hFSH molecule; (**F**) N-glycan profile for r-hFSH; (**I**) biological activity of r-hFSH in vivo. * Value below the limit of quantification.

To demonstrate the industrial suitability of the described purification process, Figure 5 shows the batch-to-batch consistency of the actual batches of the r-hFSH drug substance with regard to several quality parameters: product-related impurities (oligomeric forms, free subunits, and oxidized forms), host-cell impurities (host-cell DNA and HCP), biological activity, and glycosylation parameters (bound sugars and N-glycan profile of the r-hFSH molecule). Ten full-scale batches were included in the analysis. All batches were obtained using the same filled industrial columns (except the Sephadex G25 column, which was repacked by the new resin batch after the fifth product batch of the ten batches shown).

The specification limits for purity of every r-hFSH molecule are described in the chapter “Follitropin concentrated solution” of the European Pharmacopoeia [31]. The mean content of the impurities in the ten batches of studied r-hFSH is presented in Figure 5. Thus, the mean level of r-hFSH oligomers was 0.06% (the upper acceptance limit is 0.5%); the content of free subunits was 0.5% (the upper acceptance limit is 3%); the sum of oxidized forms of alpha- and beta-subunits of r-hFSH was 1.6% (the upper acceptance limit is 6%); and the r-hFSH concentration was 0.5 mg/mL (specified range is from 0.4 to 0.8 mg/mL) (Figure 5). The mean biological activity of r-hFSH was 13.3 kIU/mg (the estimated potency is not less than 80% and not more than 125% of the stated potency of 13.6 kIU/mg), and the N-glycan composition and sialylation hypothetical Z-index was 214 (specified range is from 177 to 233).

The host-cell-derived DNA limits are not specified for r-hFSH in [31]. Based on the WHO and FDA guidelines [32,33], the limit of residual DNA for continuous non-tumorigenic cells, such as CHO, should be less than 10 ng/dose. In the case of studied r-hFSH, the upper limit of 120 pg of DNA per mg of r-hFSH was specified as equal to 3.96 pg of host-cell DNA per dose of r-hFSH, which is less than the maximal allowed 10 ng/dose. The mean content of host-cell DNA in the ten batches of studied r-hFSH substance was 10.4 pg per mg of r-hFSH (Figure 5B).

The host-cell-derived protein content is not specified for r-hFSH in [31]. However, the maximal level should be avoided, and the upper limit can be specified as 100 ng of proteins per mg of active ingredient [34]. The mean content of host-cell proteins in analyzed r-hFSH was 2.93 ng per mg, with the specified internal upper limit of 20 ng per mg (Figure 5E).

## 4. Discussion

An estimated 100 million units of gonadotropin was used in 2016 for ovarian stimulation, and this amount is predicted to continue to increase by 6% each year; moreover, the use of gonadotropin has led to the birth of >15 million children worldwide [35]. Since the 1910s, hormones extracted from animal pituitaries and pregnant mare serums, and since the 1930s, those from human pituitaries, placenta, and urine have been used for ovulation induction in humans [16]. r-hFSH appeared in the 1990s, and it was later marketed as follitropin alpha and follitropin beta [24,36]. To date, the choice of gonadotropin for ovarian stimulation is based on availability, convenience, and costs, and further research is unlikely to identify any differences at the clinical level [37]. On the other hand, the choice of gonadotropin preparation for ovarian stimulation is influenced by the regimen of pituitary desensitization, ovarian reserve, and overall clinical assessment [38].

As a result of patent expiration of follitropin alpha in 2012, biosimilars started to appear in Europe with the aim of marketing an effective preparation at lower costs to allow more couples to gain access to assisted reproductive technology [39]. Biosimilars are not exact copies of the reference product, particularly because of the dissimilarity in the host cell line and genetic vectors used for its modification; moreover, the manufacturing and purification processes are different [39]. Therefore, the manufacturer of r-hFSH biosimilars is required to conduct Phase I and III randomized controlled trials aiming to demonstrate that the changes in research, development, and manufacturing processes do not affect the chemical identity, purity, potency, and safety of the finished product. According to the guidelines of European Medicinal Agency for r-hFSH, biosimilar products should be assessed only in comparison with the original r-hFSH in pre-clinical, pharmacokinetics, and pharmacodynamics studies [40]. It is known that r-hFSH biosimilars can differ slightly in their glycosylation and sialylation profiles [35]. The most significant differences occurred in the fractions of highly branched structures of N-glycans observed for the studied r-hFSH substance compared with the original r-hFSH [18]. In addition, the most prominent peak in the chromatograms of the sialic acids from r-hFSH biosimilar and original r-hFSH corresponded to N-acetylneuraminic acid (Neu5Ac) with 92.9% in r-hFSH substance and 75.4% in originator [18]. The rise in r-hFSH biosimilars should suggest not only a lower cost for ovarian stimulation protocol but also a safety compared with the original products. The manufacturing process of any biosimilar product should be validated to prove that both the batch-to-batch consistency of the product and the safety has to be demonstrated by clinical studies.

r-hFSH is produced in mammalian cell culture, and the production involves proper upstream (cell cultivation) and downstream (isolation from culture medium, purification, and concentration) processes. Different downstream processes, including several ultrafiltration/diafiltration steps for r-hFSH, have been previously reported. The ultrafiltration step was adopted because of the low concentration of the target protein in the culture broth. We used a previously developed r-hFSH producer cell line with a relatively high productivity [23], and thus it was necessary to include an ultrafiltration step. As a key step in the downstream process, we used immunoaffinity chromatography based on the resin containing camelid single-domain antibody fragments [25]. The resin showed selectivity for intact r-hFSH only, but not toward a single alpha or beta chain of FSH [25]. In the present study, we described its behavior in the production process. The downstream process for r-hFSH consists of an initial solvent/detergent viral inactivation step, multimodal, immunoaffinity, desalting by size exclusion, tandem ion exchange, final size exclusion chromatography, viral filtration step, and formulation. The first chromatographic step is performed using a multimodal resin which exhibits several functionalities based on ionic, hydrogen bonding, and hydrophobic interaction. This step serves for primary isolation of rh-FSH from the culture medium and concentration of the semi-product. Through highly specific antibody–antigen based interactions in immunoaffinity chromatography, rh-FSH is selectively separated from HCP. The eluate from the immunoaffinity stage requires desalting by size exclusion for subsequent ion-exchange chromatography, which separates differently charged rh-FSH variants and enables rh-FSH with proper biological activity to be yielded. The final size exclusion is aimed at purification from protein dimers, multimers, and free subunits. Herein, we present data on the stability of all five chromatographic resins used for 40 chromatographic cycles in this study, including resin cleaning and sanitization procedures. For multimodal, immunoaffinity, and ion-exchange chromatography, DBC was determined. For multimodal chromatography, the sample residence time was optimized.

In multimodal chromatography, we observed an increase in the resin capacity in the later cycles based on the elution data (Figure 2B). This increase in eluted r-hFSH can be explained by the improved binding of r-hFSH to the functional groups of the resin after several cycles of elution and regeneration or by gradual compression of the resin in the packed bed, resulting in a change in the available space in the pores of the resin.

Immunoaffinity resins are often used for purification of recombinant proteins, for example, for the purification of blood clotting factor VIII [41]. The authors of [27] have examined in detail the use of a VIIISelect resin consisting of a camelid-derived 13 kDa recombinant single domain mini-antibody ligand attached to a cross-linked agarose base matrix. The study involved the measurement of the static binding capacity, dynamic binding capacity, product recovery, impurity clearance, and adsorbent reuse. However, similar data were not available for the CaptureSelect FSH Affinity Matrix used in the process described. We determined the DBC_10%_ for the immunoaffinity resin (1.6 mg FSH/mL resin in cycle 1) and studied its behavior in reuse cycles. The increased FSH breakthrough that occurred between the 30th and the 40th cycles indicated that the binding capacity of the resin diminished to some extent after 30 use cycles, and a larger amount of FSH started to appear in the breakthrough (Figure S2). At the same time, until a capacity of 1.0 mg/mL was reached, no detectable amounts of FSH appeared in the flow-through fractions in all cycles (Figure S2). Thus, the change in the shape of the r-hFSH binding curve at the 40th cycle possibly did not indicate a drop in capacity but a change in the additional FSH binding through another mechanism (binding by other functional groups of the resin, not by the antibodies). Therefore, we used another common method to assess the capacity change by evaluating the target protein content in the eluate. The analysis of the eluate showed that the resin capacity remained the same over 40 cycles (Figure 2C,D).

It is important that the purification process is designed to maximize the removal of impurities. Affinity chromatography resins can be a source of polypeptide leachables that contaminate the target protein and are potentially immunogenic to patients. We observed a drop in the leaked antibodies level after cycle 10. We suppose that some of the immobilized antibodies lie on the external surface of the resin particles and are better exposed to the proteases which are present in the culture medium along with the other HCP, and thus this small subfraction of the antibodies is washed away from the resin in the early use cycles. In all cycles, the concentration of anti-FSH mini-antibodies in the affinity column eluate was less than 2 ng/mg FSH (Figure 3A). We also analyzed three batches of the r-hFSH drug substance for the presence of anti-FSH mini-antibodies and found that their concentration in the substance was below the quantification limit, which corresponded to <0.2 ng/mg FSH. The proportion of the contaminating fragment of the camelid antibodies among other HCP was <1% (the typical value of the HCP level in the r-hFSH drug substance was 3–10 ng/mg FSH, Figure 5E). We observed an extremely low level of HCP (10 ng/mg FSH) in the affinity column eluate in cycle 1 (Figure 3B), which might be caused by irreversible adsorption of HCP on the resin surface by residual chemically active functional groups used for the ligand–matrix conjugation. In the following cycles, the saturation of the resin was achieved, and the range of HCP in the eluate stabilized to 60–130 ng/mg FSH. As a result of the total purification process, the content of HCP in the substance was <20 ng/mg FSH. This implies that both HCP and camelid anti-FSH antibodies were eliminated from the semi-purified product during the further chromatographic stages of the purification process. All adsorption chromatography steps used in the process developed were also effective in the removal of the model viruses of various classes, together with the dedicated viral inactivation and virus filtration steps, which reduced the level of all model viruses tested by at least 12 orders of magnitude.

The purification process resulted in a sufficiently pure product with an extremely low level of host cell impurities (residual HCP and DNA, Figure 3B,E), very low product-related impurities (oligomeric forms, free chains, and oxidized forms, Figure 5A,D,G), and practically absent macromolecular process-related impurities (camelid anti-FSH mini-antibodies). Non-properly glycosylated r-hFSH molecules were removed by ion-exchange chromatography, which was confirmed by the analysis of bound sugar (Figure 5C) and N-glycan profile (Figure 5F). At least ten batches of r-hFSH, with consistent biological activity, were obtained (Figure 5I) without a change in the resin in most of the chromatography columns. The quality control results for the r-hFSH drug substance indicated that the r-hFSH production process successfully achieved upscaling, with consistent performance across batches.

## 5. Conclusions

Using the developed and optimized downstream process, this study demonstrated the possibility of obtaining r-hFSH with a batch-to-batch consistency in terms of chemical structure and biological activity.

## Figures and Tables

**Table 1:** Target protein loss at DBC_10%_ for Capto MMC resin at various residence times. Data for cycles 1–3.

**Residence Time**	2 min	1 min	40 s
**Total FSH breakthrough**	2.6%	1.0%	2.7%

**Table 2:** Leaked antibodies content in the recombinant human follicle-stimulating hormone (r-hFSH) drug substance. * value below the quantification limit (LOQ = 0.080 ng/mL).

Batch No.	Leaked Antibodies, ng/mL	Leaked Antibodies, ng/mg FSH	SD, ng/mg FSH
1	0.058 *	0.117	0.011
2	0.055 *	0.109	0.015
3	0.061 *	0.122	0.021

**Table 3:** Reduction factor (RF) for model viruses pseudorabies virus (PRV), parainfluenza virus (PIV), reovirus type-3 (REO), and encephalomyocarditis virus (EMC) in five downstream steps of r-hFSH.

Model Virus	Downstream Step															
	Solvent/Detergent Inactivation			Ion Exchange Chromatography (Capto Q + Sartobind S Membrane)			Immunoaffinity Chromatography (CaptureSelect FSH Affinity)			Multimodal Chromatography (Capto MMC)			Nanofiltration (Virosart CPV)			Total
	Run No.			Run No.			Run No.			Run No.			Run No.			
	1	2	3	1	2	3	1	2	3	1	2	3	1	2	3	
PIV	3.3	3.7	4.0	4.7	4.7	5.2	3.0	3.1	3.0	4.2	4.7	3.7	4.0	3.9	4.4	19.9
PRV	5.0	6.0	5.0	5.8	6.2	6.5	4.4	3.9	4.4	2.7	3.0	2.6	4.7	4.6	4.4	23.1
EMCV	-	-	-	1.9	3.3	0.3	3.0	2.0	3.5	2.8	3.2	3.1	5.3	4.9	4.6	12.6
REO	-	-	-	1.0	0.7	1.9	3.3	2.8	3.5	2.9	2.5	2.9	4.7	5.7	4.4	12.1

**Table 4:** Estimation of viral particles in finished product.

Model Virus	Calculation of Estimated Virus Particles/ Maximum Daily Dose of r-hFSH	Approximate Probability of One Virus Particle per of r-hFSH Doses Expected
PIV	1.1 × 10^−^^16.9^	quadrillion
PRV	1.1 × 10^−20.1^	quintillion
EMCV	1.1 × 10^−^^9.6^	billion
REO	1.1 × 10^−^^9.1^	billion

## Data Availability

All relevant data are within the manuscript and its Supplementary Material.

## Supplementary Materials

The following are available online at https://www.mdpi.com/article/10.3390/pharmaceutics14010096/s1, Figure S1: Follicle stimulating hormone (FSH) breakthrough curves for dynamic binding capacity (DBC) determination of Capto MMC resin. Figure S2: FSH breakthrough curve for DBC determination of CaptureSelect FSH Affinity Matrix. Figure S3: Load volume optimization for Sephadex G25 Medium resin. Figure S4: Optimization of ion-exchange chromatography.
