# Supplementary material for: Purification Process of a Recombinant Human Follicle Stimulating Hormone Biosimilar (Primapur®) to Yield a Pharmaceutical Product with High Batch-to-Batch Consistency
Source: Pharmaceutics. 2022 Jan 1;14(1):96. doi: 10.3390/pharmaceutics14010096 (PMC8781808; doi:10.3390/pharmaceutics14010096)
Supplement: Supplementary file 1 [file pharmaceutics-14-00096-s001.zip › pharmaceutics-1508255-supplementary.pdf]

pharmaceutics

MDPI

Supplementary Materials: Purification Process of a Recombinant Human Follicle Stimulating Hormone Biosimilar (Primapur®) to Yield a Pharmaceutical Product with High Batch-to-Batch Consistency

Maria Sinegubova, Ivan Vorobiev, Anatoly Klishin, Dmitry Eremin, Nadezhda Orlova, Natalya Orlova and Mikhail Polzikov

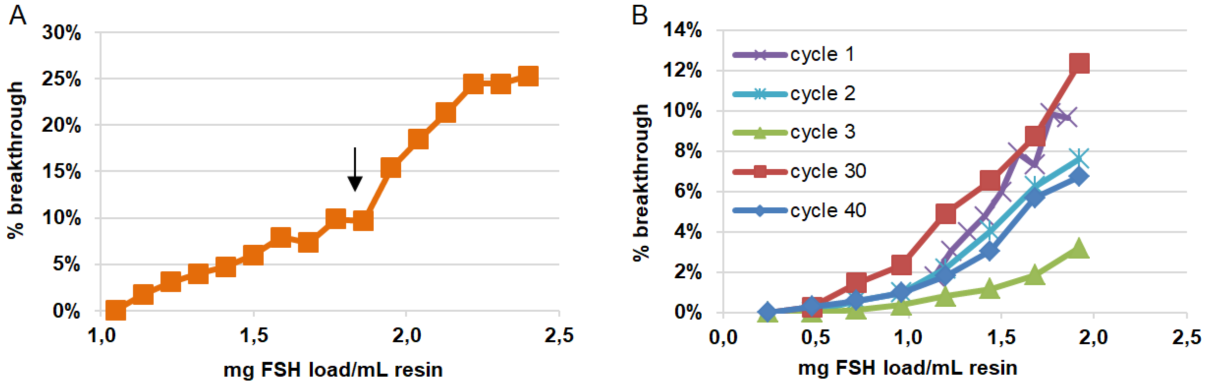

Figure S1. Follicle stimulating hormone (FSH) breakthrough curves for dynamic binding capacity (DBC) determination of Capto MMC resin. FSH concentration measured by enzyme-linked immunosorbent assay (ELISA). (**A**) Control cycle 1. Load 2.5 mg FSH/mL. Residence time 2 min, Tricorn 5/200 column, bed volume 4mL. The arrow indicates the DBC at 10% breakthrough ($DBC_{10\%}$ = 1.9 mg FSH/mL resin). (**B**) Control cycles 1, 2, 3, 30, and 40. Load 2.0 mg FSH/mL. Residence time 1 min, Tricorn 5/50 column, bed volume 1 mL.

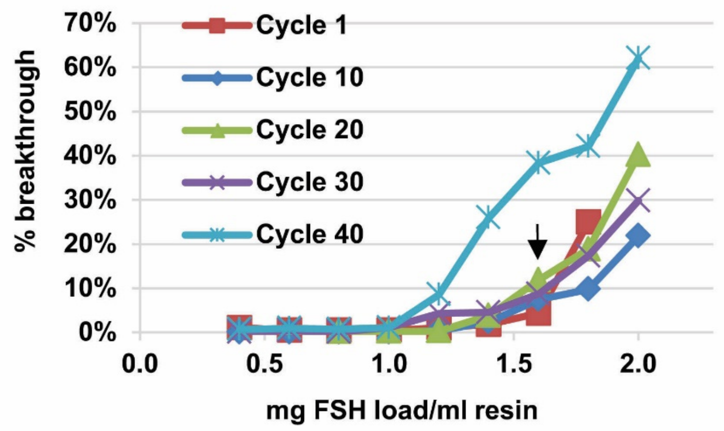

Figure S2. FSH breakthrough curve for DBC determination of CaptureSelect FSH Affinity Matrix. FSH concentration measured by ELISA. Control cycles 1, 10, 20, 30, and 40. Load 2.0 mg FSH/mL. Residence time 5 min. Tricorn 5/50 column, bed volume 1mL. The arrow indicates the DBC at 10% breakthrough ($DBC_{10\%}$ = 1.6 mg FSH/mL resin) as determined for cycles 1–30.

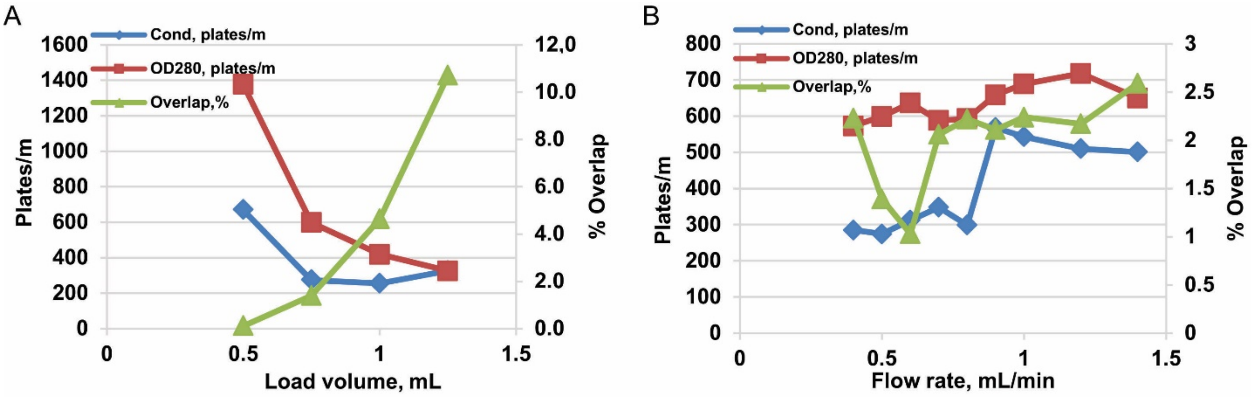

Figure S3. Load volume optimization for Sephadex G25 Medium resin. Tricorn 5/200 column, bed volume 4mL. Applied 0.5 mg/mL bovine serum albumin (BSA) in 2M $MgCl_2$. Detection in conductivity and optical density (OD) at 280 nm channels. (**A**) Peak overlapping and resolution for elution peaks under various load volumes (0.5-1.25 mL). (**B**) Peak overlapping and resolution for elution peaks under various flow rates (0.4–1.4 mL/min), load volume 0.75 mL.

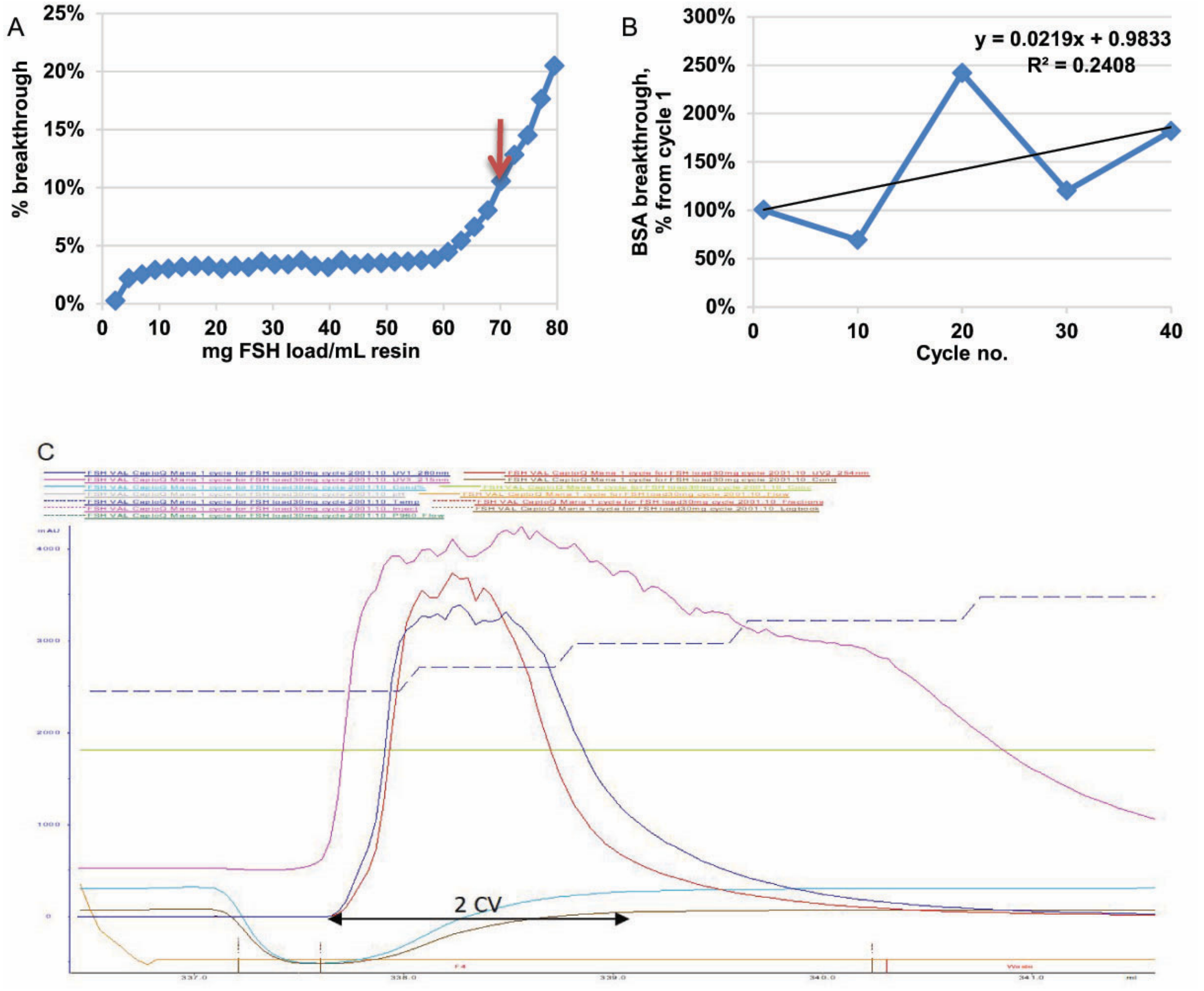

Figure S4. Optimization of ion-exchange chromatography. Tricorn 5/50 column, bed volume 1 mL, CaptoQ resin. (**A**) FSH breakthrough curve for dynamic binding capacity (DBC) determination of Capto Q resin. Load 80 mg FSH/mL resin. FSH concentration in flowthough measured by absorption at 280 nm. The arrow indicates the DBC at 10% breakthrough ($DBC_{10\%}$ = 70 mg FSH/mL resin). (**B**) Estimation of the DBC change: BSA breakthrough in control cycles in absorbance channel at 280 nm. Load 320 mg BSA/mL resin. (**C**) Estimation of FSH elution peak volume. Loaded 30 mg FSH/mL. The elution peak volume is 2 mL, or 2 column volumes (CV, arrowed). The criterion for stopping the collection of the eluate was 5% of the peak height in absorbance channel.
